# A Prospective Analysis of the Simplified Student Sight Savers Program on Open-Angle Glaucoma Cost Burden in Underserved Communities

**DOI:** 10.3390/jcm11102903

**Published:** 2022-05-20

**Authors:** Justin T. Bradshaw, Telyn Peterson, Lawsen M. Parker, Zeke Richards, Chad J. Skidmore, Kevin Brighton, Maxton W. Muir, Alexandra Moody, Andrew Collyer, Isain Zapata, Amanda E. Brooks, Marcos Reyes

**Affiliations:** 1 Rocky Vista University College of Osteopathic Medicine—Southern Utah, Ivins, UT 84738, USA; telyn.peterson@rvu.edu (T.P.); lawsen.parker@rvu.edu (L.M.P.); zeke.richards@rvu.edu (Z.R.); chad.skidmore@rvu.edu (C.J.S.); kevin.brighton@rvu.edu (K.B.); maxton.muir@rvu.edu (M.W.M.); alexandra.moody@rvu.edu (A.M.); andrew.collyer@rvu.edu (A.C.); abrooks@rvu.edu (A.E.B.); 2 Rocky Vista University College of Osteopathic Medicine—Colorado, Parker, CO 80134, USA; izapata@rvu.edu; 3 St. George Eye Center, St. George, UT 84790, USA

**Keywords:** glaucoma, glaucoma screening, student-led, elevated IOP, tonometer, glaucoma cost, underserved communities

## Abstract

(1) Background: Glaucoma is a leading cause of irreversible blindness worldwide. Unfortunately, no noticeable symptoms exist until mid- to late-stage glaucoma, leading to substantial costs to the patient and the healthcare system. (2) Methods: The Student Sight Savers Program, an initiative started at Johns Hopkins University, was designed to meet the needs of community screening for glaucoma. Several medical students at the Rocky Vista University in Saint George, Utah, were trained, and screened patients at local fairs and gathering places using a modified version of this program. Patients found to have elevated pressure (>21 mmHg) or other ocular abnormalities were referred for an ophthalmological examination. (3) Results: Individuals from medically underserved areas/populations (MUA/Ps) were nearly three times as likely to have elevated intraocular pressure as individuals not in underserved areas (*p* = 0.0141). A further analysis demonstrates that medical students can help reduce medical costs for patients and the healthcare system by providing referrals to ophthalmologists and reaching populations that are not usually screened for glaucoma. (4) Conclusions: Allowing medical students to perform community-based glaucoma screening events in MUA/Ps using handheld tonometers may decrease the cost burden associated with late diagnosis, and raise awareness about glaucoma, especially in underserved populations.

## 1. Introduction

Glaucoma is a leading cause of irreversible blindness worldwide. Although there are multiple risk factors associated with glaucoma, an elevated intraocular pressure (IOP) is a main risk factor [1]. Since IOP measurements varying from high to low can be present in each glaucoma patient depending on their own anatomical characteristics [2], defining a problematic elevated IOP is difficult for the general population. However, elevated IOP is generally characterized by the National Eye Institute as being greater than 21 mmHg in most settings [3]. The staging of glaucoma is appropriately performed using the six-stage system of the Bascom Palmer Glaucoma Staging System, which centralizes on a basis of the Humphrey visual field test [4]. While several screening tests are involved in evaluating people at risk for glaucoma, eye pressure checks using handheld tonometers are an easy and effective way to find elevated IOP in its early stages, before extensive sight-limiting nerve damage has occurred [5]. While traditional techniques to detect elevated IOP, a crucial step in disease progression, include the use of a slit lamp or indirect ophthalmoscope, the use of handheld tonometers to detect IOP requires less technical skill and training; however, historically, tonometers have only been used in ophthalmology or inpatient settings, and not widely used in the primary care setting [6]. Training medical students to use a handheld tonometer may have a significant impact on the situation, allowing more community-based screening opportunities. A recent study compared the effectiveness of eye pressure screenings in a primary care setting versus a community setting. Although both settings are beneficial in promoting ophthalmic follow-up, it has been demonstrated that primary care settings lead to a higher likelihood of follow-up with an ophthalmologist [7]. Additionally, providing training on the benefit and use of ocular tonometry to medical students can increase the likelihood that they use tonometers in their future practice, regardless of specialty. Studies have been conducted to show that tonometer training through immersive experiences provides a useful skill for future use in emergency departments and primary care settings for students not primarily interested in ophthalmology as a future career [8].

While the 2014 United States Preventive Services Task Force has no guidelines on routine glaucoma screening due to a lack of data, routine screening by students has not been studied at length. This ongoing study examines the value of medical student-run IOP screening booths as an option for community-based glaucoma prevention, especially in people who reside in medically underserved areas/populations (MUA/Ps). Allowing medical students to perform community-based glaucoma screening events using handheld tonometers can not only decrease the cost burden associated with late diagnosis, but perhaps raise awareness of glaucoma, especially in underserved populations [9,10].

### Underserved Populations

Within the United States, there are geographical areas designated as regions that have a shortage of medical services, where the population is underserved. These designations can be further described as health professional shortage areas (HPSAs) and/or medically underserved areas/populations (MUA/Ps). According to the United States Department of Health & Human Services, HPSAs are identified as areas, population groups, or facilities within the United States that are experiencing a shortage of healthcare professionals, whereas MUA/Ps are identified as areas or populations with a shortage of primary care health services within a geographic area or a specific population subset [11]. The MUA/P designation includes populations that face economic, cultural, or language barriers to health care, such as people experiencing homelessness, a low income, and/or lack of other resources [11].

Medically underserved areas/populations are often found within rural regions. Communities within these rural regions face higher poverty rates, lower educational attainment, a lack of transportation, a higher proportion of elderly people, and a lack of access to health services [12]. Rural areas also have a higher prevalence of several chronic diseases, such as coronary heart disease and diabetes [12]. A recent study found that reductions in diabetes mortality are lagging in rural areas, especially in rural areas in the south [13]. Importantly, some of the main risk factors for glaucoma include old age, diabetes, and hypertension, some of the same diseases that disproportionately affect individuals in rural areas. Therefore, MUA/Ps may be at an increased risk of developing advanced glaucoma with significant visual loss, justifying the need to increase awareness and screening in these areas.

## 2. Materials and Methods

**Figure 1:**
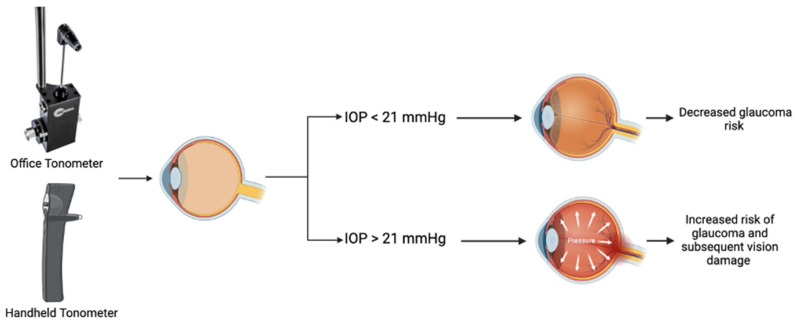
IOP threshold used for assessing risk in participants.

The portable I-care tonometer was found to be an easy and reliable tool to use in community settings in previous studies [5,14,15]. Medical students underwent training in proper use of a handheld tonometer, which consisted of training in maintaining the device perpendicular to the eye when taking measurements, sanitation of the device between participants, and recording findings for each eye. The threshold for assessing risk in participants was an IOP measurement greater than 21 mmHg (Figure 1).

**Figure 2:**
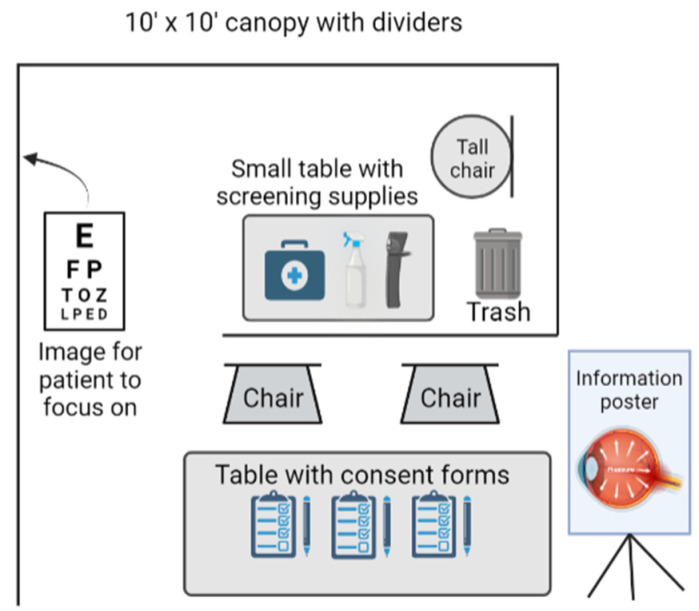
Schematic of the setup used for IOP screenings.

First- and second-year medical students from Rocky Vista University screened individuals attending local community events in Southern Utah in a semi-private screening station. No prior advertising was used to recruit people to the booth at the event, other than a poster on the booth that stated, “Free Eye Pressure Screenings”. Participants who voluntarily approached the booth at random, chose to participate in the study, and did not fall under the exclusion criteria were included in the research. Participants were excluded if they were under 40 years of age, according to the recommendations of recent studies, which highlighted the increased risk of glaucoma in older individuals [1]. Additionally, participants who wore contact lenses in both eyes were excluded due to the lack of proper lens solution and a hand washing station to remove the contact lens, although recent research that came out during this study suggested good reliability of IOP measurements over contact lenses of different materials and thickness profiles while using rebound tonometry [16]. Two individuals with contact lenses in one eye were screened in the eye without the contact lens. The screening station consisted of a booth with a draped semi-private area in the back where participants’ IOPs were measured. At the front of the screening station was a table where participants signed a consent and liability form for screenings, as well as a research agreement form (Figure 2).

**Figure 3:**
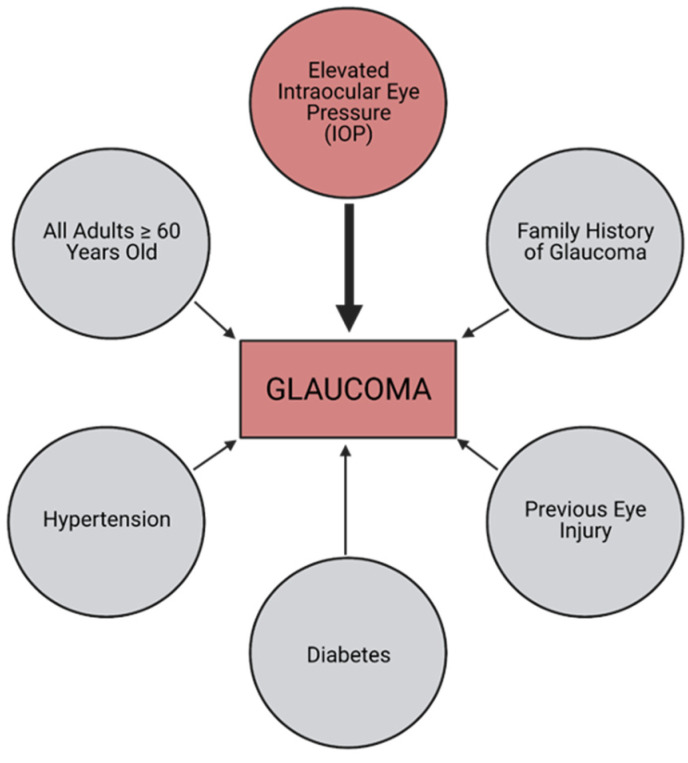
Common risk factors for developing glaucoma.

**Figure 4:**
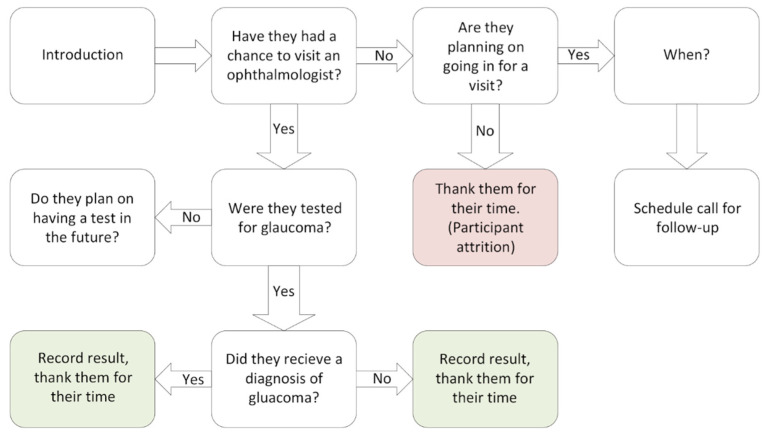
Flow chart of questions asked during the telephone call follow-up with participants.

Participants provided demographic information at the time of screening, including age, ethnicity, residence address, and phone number, to allow for the evaluation of the community outreach performed and follow-up. Participant addresses were used to determine if the participant was a resident of an HPSA or MUA/P according to the Health Resources & Service Administration (HRSA) website [17]. After completing the consent form and demographic information, participants were brought back to the semi-private screening area, and a brief medical and family history with respect to ocular diseases was collected. Regardless of the test results, participants were encouraged to see an ophthalmologist regularly, especially if they had an abnormal IOP measurement greater than 21 mmHg, or other underlying risk factor for glaucoma, as determined by the National Eye Institute [3] (Figure 3). Within six months after screening, individuals were contacted by phone and interviewed to see what actions they took in relation to their screenings (Figure 4).

## 3. Results

**Figure 5:**
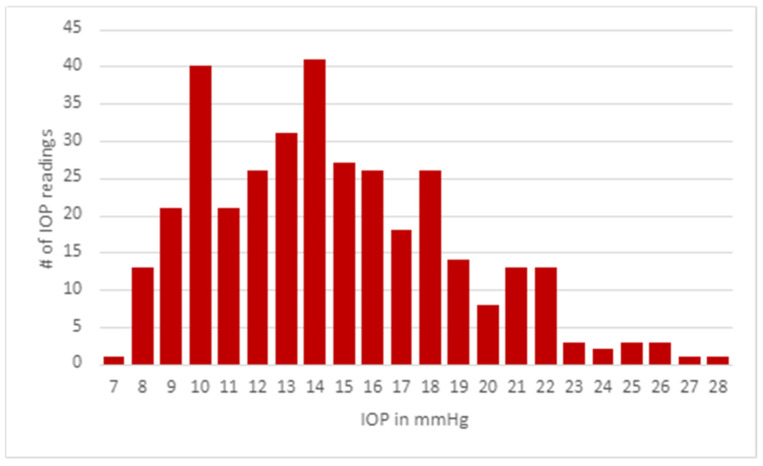
Distribution of IOP measurements of participants.

During 57 h of screening at local community events, including conferences and farmers markets, 352 eyes from 177 participants of an average age of 62.5 years had their eye pressures checked by medical students using a handheld I-care tonometer (Figure 5). In addition to IOP screenings, the booth spread awareness of glaucoma through the use of flyers. The overall average IOP including both eyes was 14.56 mmHg (STDEV = 4.24). The mean IOP reading for the right eye was 14.63 mmHg (*n* = 176; SD = 4.177 mmHg) and the mean IOP reading for the left eye was 14.49 mmHg (*n* = 176; SD = 4.316 mmHg). There were no significant differences between the overall average IOP measurements of the right and left eyes (*p* = 0.7573).

From the total participants, 102 were women and 75 were men. The women who were screened had slightly higher IOP measurements (14.78 mmHg; STDEV = 4.37 mmHg) than the male participants (14.26 mmHg; STDEV = 4.05), but this finding was not statistically significant (*p* = 0.4209). In total, 14 of the 102 female participants (13.7%) had IOP measurements greater than 21 mmHg, while only 7 of the 75 male participants (9.3%) had IOP measurements greater than 21 mmHg, but these findings were also not significant (*p* = 0.3724). Contrary to the trends in these measurements, most studies with larger sample sizes suggest that men tend to have higher IOP than women [18,19].

**Figure 6:**
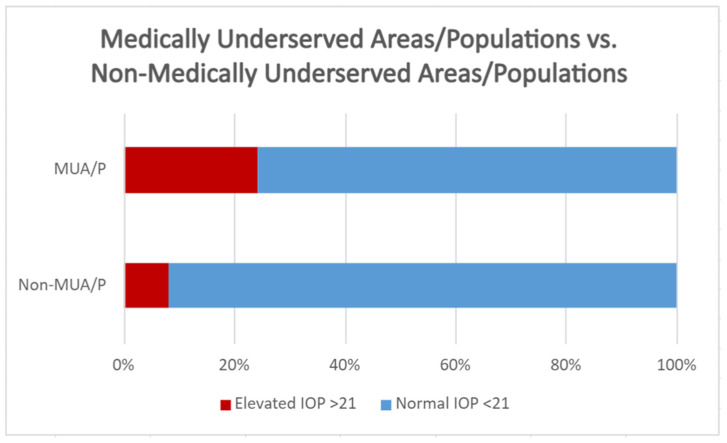
Percentage of IOP > 21 mmHg in MUA/Ps vs. non-MUA/Ps.

Of the 177 participants, 21 had elevated pressures (IOP > 21 mmHg). Twenty of the twenty-one participants that had elevated pressures had addresses in areas of primary care healthcare professional shortage areas (HPSAs). In total, 43 of the 177 individuals indicated that they were from medically underserved areas/populations (MUA/Ps) as defined by the address. Of the 43 who were from MUA/Ps, 10 had elevated IOP (24.3%). Of those who were not from an MUA/P, 11 of 134 had elevated IOP (8.21%). These results demonstrated that individuals classified as MUA/Ps were almost three times as likely to have high IOP (*p* = 0.0041). Furthermore, when comparing the mean IOP of individuals in an MUA/P (15.6 mmHg, STDEV = 4.45) with those not in an MUA/P (14.3 mmHg, STDEV = 4.15), it was found that MUA/P individuals had a higher IOP (*p* = 0.0141) (Figure 6).

## 4. Discussion

The use of a low-cost glaucoma screening by medical students can be beneficial to medically underserved areas. Since most medical schools in the United States have both the resources and the responsibility to provide rural clinical experiences to their students [20], medical student-run screenings are a viable option to reach underserved populations. This is of particular importance in light of our study results that showed an increased incidence of elevated IOP in MUA/Ps, which could lead to the subsequent development of glaucoma. While the underlying cause is not immediately clear, it may be due to the discrepancy in risk factors when comparing urban and rural populations, such as the higher incidence of diabetes and hypertension in rural areas [12]. Nevertheless, individuals from MUA/Ps should be screened more frequently to assess the IOP and glaucoma risk, necessitating out-of-the-box solutions to provide access.

Training medical students through immersive experiences to correctly use handheld tonometers can increase the likelihood that they use them in their future practice (practices that include emergency departments and primary care settings) [7]. Medical students interested in primary care and primary care providers are better suited to reaching underserved areas and populations, due to the decreased likelihood of rural ophthalmic professionals, especially those who sub-specialize. Using these described methods provides medical students studying in rural areas with the opportunity to volunteer during medical school and find individuals with an increased risk of glaucoma.

### 4.1. Cost Burden

Like any chronic disease, the cost of glaucoma care can become a burden for both the patient and the healthcare system. The cost of treating and monitoring glaucoma is frequently much higher in the advanced stages of the disease compared to the earlier stages [21]. Through early detection and diagnosis, it has been established that the overall cost of glaucoma can be mitigated by preventing the disease from progressing [9].

**Figure 7:**
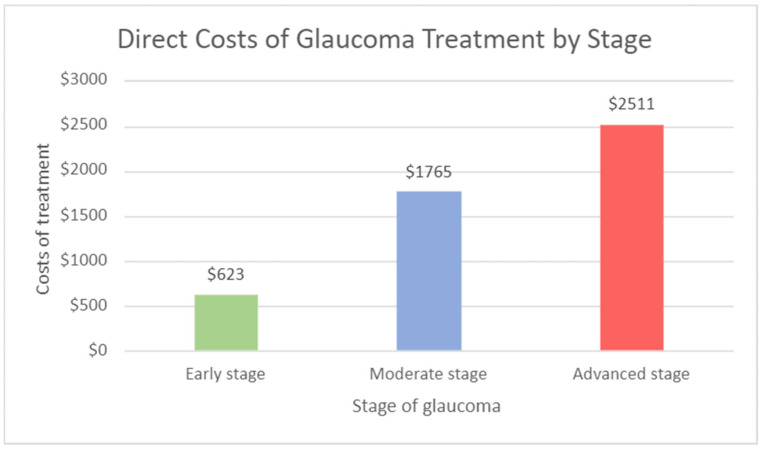
Direct cost of glaucoma treatment by stage.

Direct costs of glaucoma include ophthalmology visits, surgeries, and medication use. In its early stages, direct costs for glaucoma average USD 623 per patient per year. A drastic increase in advanced-stage management has been observed, with costs averaging USD 2511 per patient per year [10]. An annual average of USD 1888 can be saved if glaucoma is diagnosed and controlled in its early stages rather than in its advanced stage (Figure 7). In addition to the direct costs as outlined above, patients with advanced-stage glaucoma also have costs associated with low-vision care and vision rehabilitation [10]. The early detection of glaucoma can prevent vision loss, which would eliminate these costs for the patient. 

### 4.2. Drawbacks/Limitations

While this study represents a significant opportunity to address a significant healthcare burden and narrow the gap in ophthalmological care, it was not without limitations. First, the results were limited to the small, medically underserved geographic region used for screening, and their general utility remains to be explored. Two of the four venues for screening occurred in health-based events, where many people are health conscious. Running screenings in churches, grocery stores, and other places that are not correlated with visits by only health-conscious consumers may have changed the demographics of the sample population. Additionally, we did not factor in the role of optometrists in this research and how they can provide screenings and awareness to underserved rural populations; however, this does not negate the benefits provided by student training.

### 4.3. Future Research

The tools for glaucoma screening are continuing to be developed. Recently, a newly developed low-cost, smart-phone-based mobile virtual perimetry (MVP) frequency doubling technology was found to produce results comparable to the traditional Humphrey Zeiss frequency doubling technology for visual field testing, which also may be used as an easily accessible screening tool for glaucoma [22], providing yet another easy-to-access technology that students can use to provide community-based glaucoma screenings. This should be examined in future studies as an important adjunct tool for student-run screening and community outreach.

To date, there have been no studies that demonstrate the long-term use of ocular tonometry. It is well established that the study of ophthalmology is increasingly deficient during medical school as the overall curriculum expands. Future studies should consider assessing the use of tonometry to determine the prevalence of eye pressure measurement outside of the emergency room and clinics primarily focused on eye care.

## 5. Conclusions

The application of handheld tonometers to allow medical students and primary care offices to provide low-cost glaucoma screening procedures is an effective way to reach underserved populations, many of whom do not have access to regular ophthalmic care, while also preventing increased costs for both the patient and the healthcare system. If this procedure becomes standardized, medical students across the country, along with primary care providers, would be able to replicate these results in their own surrounding communities, resulting in glaucoma detection in its early stages, thus preventing permanent blindness and excess costs. 

## Data Availability

Contact Marcos Reyes to access datasets analyzed and generated during the study.
